# Immunomodulatory activity of polysaccharides isolated from *Clerodendrum splendens*: Beneficial effects in experimental autoimmune encephalomyelitis

**DOI:** 10.1186/1472-6882-13-149

**Published:** 2013-06-28

**Authors:** Koffi Kouakou, Igor A Schepetkin, SangMu Jun, Liliya N Kirpotina, Ahoua Yapi, Daria S Khramova, David W Pascual, Yury S Ovodov, Mark A Jutila, Mark T Quinn

**Affiliations:** 1 Department of Immunology and Infectious Diseases, Montana State University, Bozeman, MT 59717, USA; 2 Laboratoire d’Endocrinologie et Biologie de la Reproduction, Université de Cocody-Abidjan, Abidjan, Côte d’Ivoire; 3 Department of Infectious Diseases and Pathology, University of Florida, Gainesville, FL 32611, USA; 4 Institut National de la Santé Public, Abidjan, Côte d’Ivoire; 5 Institute of Physiology, Komi Science Centre, The Urals Branch of the Russian Academy of Sciences, Syktyvkar, Russia

**Keywords:** *Clerodendrum splendens*, Polysaccharide, Macrophage, Cytokine, Immunomodulation

## Abstract

**Background:**

Extracts of leaves from *Clerodendrum* have been used for centuries to treat a variety of medicinal problems in tropical Africa. However, little is known about the high-molecular weight active components conferring therapeutic properties to these extracts.

**Methods:**

Polysaccharides from the leaves of *Clerodendrum splendens* were extracted and fractionated by ion exchange and size-exclusion chromatography. Molecular weight determination, sugar analysis, degree of methyl esterification, and other chemical characterization of the fractions were performed. Immunomodulatory activity of the fractions was evaluated by determining their ability to induce monocyte/macrophage nitric oxide (NO), cytokine production, and mitogen-activated protein kinase (MAPK) phosphorylation. Experimental autoimmune encephalomyelitis (EAE) was induced in C57BL/6 mice, and severity of EAE was monitored in mice treated with intraperitoneal (*i.p.*) injections of the most active polysaccharide fraction. Lymph nodes (LN) and spleen were harvested, and levels of cytokines in supernatants from LN cells and splenocytes challenged with myelin oligodendrocyte glycoprotein peptide were determined.

**Results:**

Fractions containing type II arabinogalactan had potent immunomodulatory activity. Specifically, the high-molecular weight sub-fraction **CSP-AU1** (average of 38.5 kDa) induced NO and cytokine [interleukin (IL)-1α, -1β, -6, -10, tumor necrosis factor (TNF; designated previously as TNF-α), and granulocyte macrophage-colony stimulating factor (GM-CSF)] production by human peripheral blood mononuclear cells (PBMCs) and monocyte/macrophages. **CSP-AU1-**induced secretion of TNF was prevented by Toll-like receptor 4 (TLR4) antagonist LPS-RS, indicating a role for TLR4 signaling. Treatment with **CSP-AU1** also induced phosphorylation of a number of MAPKs in human PBMC and activated AP-1/NF-κB. *In vivo* treatment of mice with **CSP-AU1** and **CSP-NU1** resulted in increased serum IL-6, IL-10, TNF, monocyte chemoattractant protein-1 (MCP-1), macrophage inflammatory protein (MIP)-1α/CCL3, and MIP-1β/CCL4. **CSP-AU1** treatment of mice with EAE (50 mg/kg, *i.p.*, daily, 13 days) resulted in significantly reduced disease severity in this experimental model of multiple sclerosis. Levels of IL-13, TNF, interferon (IFN)-γ, IL-17, and GM-CSF were also significantly decreased, whereas transforming growth factor (TGF)-β was increased in LN cells from **CSP-AU1**-treated EAE mice.

**Conclusions:**

Polysaccharide **CSP-AU1** is a potent natural innate immunomodulator with a broad spectrum of agonist activity *in vitro* and immunosupressive properties after chronic administration *in vivo*.

## Background

Natural products comprise one of the most popular sources of complementary and alternative medicines for treating inflammatory and immune disorders [1]. While the mechanisms of action of many plant products remain to be elucidated, further study in this area is essential for the identification of novel therapeutics with immunomodulatory activity [2]. *Clerodendrum splendens* belongs to the family of Lamiaceae and the genus Clerodendrum, consisting of about 400 herbs, vines, shrubs, and trees of the tropics, many of which are grown as garden plants [3-5]. The plant is a climbing evergreen bush with attractive red flowers produced during the dry seasons of the year and it is very widely distributed in tropical and subtropical regions of the world [3]. Extracts of roots, leaves, and bark from *C. splendens* are used in traditional medicine to treat malaria, coughs, buboes, venereal infections, including gonorrhea and syphilis, skin diseases, ulcers, rheumatism, asthma, and uterine fibroid [3,6,7]. Leaves of this plant have also been used to treat various skin diseases and as wound-healing medicine [4]. For example, the leaves of *C. splendens* have been formulated into lotion and applied to bruises and sores, and dry powdered leaves have also been applied to blisters caused by burns [4].

Antimicrobial and wound healing properties of chloroform and methanol extracts from *C. splendens* have been confirmed in recent pharmacological studies [8,9]. The volatile oil of *C. splendens*, prepared by extraction of fresh flowers with *n*-hexane, was found to be active against S*taphylococcus aureus* and *Candida albicans*[10]. Antipyretic and anti-inflammatory effects of *C. splendens* extracts have also been described [11]. *C. splendens* has been extensively investigated, and a number of chemical constituents, including steroids, terpenoids, flavonoids, volatile constituents, cyanogenic glycoside, alkaloids, tannins, saponins, and phenolic compounds have been isolated [3,7,12-14].

Although there are many publications describing the chemical structure and bioactive effects of small molecules isolated from *C. splendens*, little is known about the immunomodulatory effects of polysaccharides extracted from the leaves of this plant. Since decoction in water is the most common mode of preparation, and the most common modes of administration are oral and local application, we hypothesized that polysaccharides extracted from *C. splendens* could have immunomodulatory properties and contribute to the therapeutic effects of extracts from this plant. To address this question, we fractionated water-soluble polysaccharides from leaves of *C. splendens* and evaluated their immunomodulatory activities. We found that the most active Clerodendrum polysaccharide fractions contained type II arabinogalactan and had potent immunomodulatory activity *in vitro* and *in vivo*. To investigate the molecular mechanism responsible for the polysaccharide-induced immunomodulatory responses, the roles of signaling pathways including mitogen-activated protein kinase (MAPK) phosphorylation and transcription factors activating protein-1 (AP-1) and nuclear factor κB (NF-κB) were evaluated.

## Methods

### Plant material

Leaves of *C. splendens* were collected in the Bingerville areas of Cote d’Ivoire. This plant was identified and authenticated by Dr. Aké-Assi, Emeritus Professor of Botany, using voucher specimens deposited at various periods, including voucher specimen #16877, deposited at the National Herbarium of the National Centre of Floristique of the University of Cocody-Abidjan. Parts of plants tested were air-dried for 7–10 days at room temperature away from direct sunlight and powdered under laboratory conditions.

### Polysaccharide fractionation

Ground leaves (500 g) were extracted with 3 L boiling distilled H_2_O for 1 hr, and the aqueous extracts were centrifuged at 2,500 × g for 15 min. A four-fold volume of ethanol was added to each supernatant to precipitate the polysaccharides overnight at 4°C. The precipitates were pelleted by centrifugation, dissolved in distilled H_2_O, centrifuged at 8,000 × g for 1 hr, and re-precipitated with a four-fold volume of ethanol. The supernatants were re-dissolved in distilled H_2_O and filtered through a 0.22 μm filter and concentrated in an Amicon concentrator with a 1 kDa PLAC membrane (Millipore, Biillerica, MA) to obtain crude extracts. The crude polysaccharide extracts were further purified using ion-exchange chromatography on a DEAE-cellulose column equilibrated with 0.05 M Tris–HCl buffer (pH 8.0). For each fractionation, the column was eluted with equilibration buffer to obtain the crude neutral polysaccharide fraction. The bound material was eluted with equilibration buffer containing 2 M NaCl. The eluates were concentrated in an Amicon concentrator with a 1 kDa PLAC membrane to obtain the crude neutral (**CSP-N**) and acidic (**CSP-A**) polysaccharide fractions. These fractions were further fractionated on Diaion HP-20 absorbent resin column (2.5 × 20 cm). For each fractionation, the column was eluted with distilled H_2_O, and the eluates were lyophilized to obtain unbound fractions (designated as **CSP-NU** and **CSP-AU**). Diaion-bound polysaccharides were eluted with methanol and dried to obtain fractions **CSP-NB** and **CSP-AB**.

Fractions **CSP-AU** and **CSP-NU** were further sub-fractionated by size-exclusion chromatography on a Sepharose-6B column (2.5×95 cm) eluted with distilled H_2_O at a flow rate of 21 ml/hr. The carbohydrate elution profile was determined by the phenol-H_2_SO_4_ method, modified to a microplate format [15], and absorbance was measured at 488 nm using a SpectraMax Plus microplate reader (Molecular Devices, Palo Alto, CA). The polyphenol elution profile was determined by the Folin-Ciocalteu assay (see below). The relevant fractions were pooled and concentrated. Buffer salts were removed from the polysaccharide samples by repeated (6×) concentration in an Amicon concentrator (1 kDa cut-off PLAC membrane) and dilution with a 10-fold volume of distilled H_2_O. For analysis of biological activity, the fractions were diluted in Hanks balanced-salt solution without Ca^2+^ and Mg^2+^ (HBSS^-^) to a concentration of 5 mg/ml and filtered through sterile 0.22 μm filters.

### Characterization of polysaccharide fractions

Average molecular weights of the polysaccharide fractions were determined by high performance size-exclusion chromatography (HP-SEC) using a Shimadzu Class VP HPC and Shodex OHpak SB-804 HQ column (8 mm × 300 mm) eluted with 50 mM sodium citrate buffer, pH 7.5, containing 0.15 M NaCl and 0.01% NaN_3_ at a flow rate of 0.3 ml/min. Peaks were detected using a refractive index detector (RID-10A; Shimadzu, Torrance, CA). Average molecular weights of the polysaccharide fractions were estimated by comparison with retention times of pullulan standards P-100, 50, 20, 10, and 5 (Phenomenex, Torrance, CA), which have molecular weights of 112, 47.3, 22.8, 11.8, and 5.9 kDa, respectively. Reproducibility of the retention times was typically >98%.

The presence of arabinogalactan in the samples was detected by single radial gel diffusion in 1% agarose gels containing 100 μg/ml β-glucosyl Yariv reagent, which selectively interacts with and precipitates compounds containing type II arabinogalactan structures [16]. Four μl of polysaccharide samples (10 mg/ml; w/v) were loaded into the wells, and the samples were incubated at 25°C for 24 hr in a humid atmosphere. A positive reaction was indicated by a reddish circle (halo) around the well, and arabic gum (4 mg/ml) (Fluka Chemie GmbH, Germany) served as a positive control.

The total amount of polyphenols in the polysaccharide fractions was determined by the Folin-Ciocalteu assay [17]. Briefly, 250 μL of Folin’s phenol reagent was added to the samples dissolved in 500 μL distilled water. After 3 min at room temperature, 1.25 mL of 20% sodium carbonate was added, mixed, and the mixture was allowed to stand for 40 min. The absorbance was measured at 750 nm in a SpectraMax Plus microplate reader. Tannic acid from *Rhus semialata* (Sigma-Aldrich, St. Louis, MO) was used to generate a standard curve.

For monosaccharide analysis, the polysaccharide fractions were lyophilized and submitted for analysis to the Oklahoma Center for Glycobiology Analytical Core Lab (Oklahoma City, OK). The polysaccharide samples or background blanks were subjected to methanolysis (methanolic 2 M HCl, 16 hr, 80°C), followed by acid hydrolysis (2 M trifluoroacetic acid, 4 hr, 100°C), and the resulting monosaccharide mixtures were analyzed by high-performance anion-exchange chromatography with pulsed amperometric detection (HPAEC-PAD) on a Dionex DX-600 HPAEC system equipped with an ED50 detector (Dionex Corporation, Sunnyvale, CA). The samples were separated on a Dionex CarboPac PA20 column eluted isocratically with 12 mM NaOH at a flow rate of 1 ml/min at 22°C. For analysis of uronic acids, the column was eluted with 10 mM NaOH for 20 min, followed by a gradient of 100 mM NaOH/150 mM sodium acetate (0–100% in 45 min). Background signals were subtracted from all samples, and individual components were quantified based on electrochemical detection relative to known standards.

The content of methoxyl groups was determined using the calibration plot for methanol, as previously described [18]. Photocolorimetry was carried out at 412 nm. The degree of methyl esterification was calculated as the ratio of the molar amount of methanol to the molar amount of uronic acids (galacturonic and glucuronic acids) and is expressed as a molar percentage. M.W., molecular weight.

### Determination of endotoxin contamination

Analyses of endotoxin concentration were performed via the kinetic method with Limulus Amebocyte Lysate (LAL) kit (GenScript, Piscataway, NJ), and samples were monitored using a SpectraMax Plus microplate reader.

### Cell culture

Murine macrophage J774.A1 cells were cultured in DMEM supplemented with 10% (v/v) heat-inactivated, endotoxin-free fetal bovine serum (FBS; Atlanta Biologicals, Atlanta, GA), 100 μg/ml streptomycin, and 100 U/ml penicillin. Cells were grown to confluence in sterile tissue culture flasks and gently detached by scraping. Human monocyte-macrophage MonoMac-6 cells (DSMZ, Germany) were grown in RPMI 1640 supplemented with 10% (v/v) endotoxin-free FBS, 10 μg/ml bovine insulin, 100 μg/ml streptomycin, and 100 U/ml penicillin. Human monocytic THP-1Blue cells obtained from InvivoGen (San Diego, CA) were cultured in RPMI 1640 medium supplemented with 10% (v/v) endotoxin-free FBS, 100 μg/ml streptomycin, 100 U/ml penicillin, 100 μg/ml zeocin, and 10 μg/ml blasticidin S. These cells are stably transfected with a secreted embryonic alkaline phosphatase gene that is under the control of a promoter inducible by AP-1/NF-κB.

PBMCs were purified from human blood using dextran sedimentation, followed by Histopaque 1077 gradient separation and hypotonic lysis of erythrocytes. Blood was collected from healthy donors in accordance with a protocol approved by the Institutional Review Board at Montana State University, and informed consent was obtained from all donors for the use of their blood in this study. PBMCs were cultured at 37°C in a humidified atmosphere containing 5% CO_2_. Cell number and viability were assessed microscopically using trypan blue exclusion.

### Analysis of nitric oxide (NO) production

J774.A1 cells were plated at a density of 1.5 × 10^5^ cells/well in a final volume of 200 μl in 96-well flat-bottom tissue culture plates and incubated in medium alone or medium containing various concentrations of polysaccharide fractions or lipopolysaccharide (LPS) from *Escherichia coli* K-235 (Sigma Chemical Co., St. Louis, MO) as a positive control. Cells were incubated at 37°C in the presence of 5% CO_2_ for 24 hr, and 100 μl of the cell culture supernatants were removed and analyzed for nitrite using a colorimetric method with NaNO_2_ as the standard. Briefly, supernatants were mixed with an equal volume of Griess reagent, which was prepared by mixing one part of 0.1% (w/v) *N*-(1-naphthyl)ethylenediamine with one part of 1% (w/v) sulfanilamide in 5% phosphoric acid. After 20 min, absorbance was measured at 540 nm using a SpectraMax Plus microplate reader.

### Determination of cytokine production

J774.A1 cells, human PBMCs, or human MonoMac-6 monocytic cells were incubated for 24 hr in culture medium supplemented with 3% (v/v) endotoxin-free FBS, with or without polysaccharide fractions or LPS as a positive control. Concanavalin A (Con A) from *Canavalia ensiformis* (Calbiochem, San Diego, CA) was also used as positive control in some experiments. Cells were plated in 96-well plates at a density of 2×10^5^ cells in 100 μL per well and incubated for 24 hr. Enzyme-linked immunosorbent assay (ELISA) kits for human tumor necrosis factor (TNF) and granulocyte macrophage-colony stimulating factor (GM-CSF), or human and mouse interleukin (IL)-6 (all from Biolegend) were used to test cytokine protein levels in the cell supernatants. Where indicated, PBMCs were pretreated with or without TLR4 antagonist LPS-RS (LPS from *Rhodobacter sphaeroides*; Invivogen, San Diego, CA) for 30 min, followed by treatment with or without polysaccharide sub-fraction **CSP-AU1**, LPS, or 200 nM of phorbol-12-myristate-13-acetate (PMA, Sigma-Aldrich, St. Louis, MO) for 24 hr, and TNF levels were evaluated in cell supernatants using an ELISA kit. A human cytokine Multi-Analyte ELISArray™ Kit (SABiosciences Corporation; Frederick, MD) was also utilized to evaluate various cytokines (IL-4, IL-6, IL-8, IL-10, IL-12, IL-17A, IFN-γ, TNF, and GM-CSF) in supernatants of PBMCs.

### Analysis of AP-1/NF-κB activation

Activation of AP-1/NF-κB transcription factors was measured using an alkaline phosphatase reporter gene assay in THP-1Blue cells. Polysaccharide fractions or LPS (100 ng/ml) were added, and the cells (2 × 10^5^ cells/well) were incubated for 24 hr. Alkaline phosphatase activity was measured in cell supernatants using QUANTI-Blue mix (InvivoGen). Activation of AP-1/NF-κB is reported as absorbance at 655 nm and compared with samples treated with positive control LPS.

### Cytotoxicity assay

Cytotoxicity was analyzed with a CellTiter-Glo Luminescent Cell Viability Assay Kit (Promega, Madison, WI), according to the manufacturer’s protocol. Following treatment, the cells were allowed to equilibrate to room temperature for 30 min, substrate was added, and the samples were analyzed with a Fluoroscan Ascent FL microplate reader.

### Mitogen-activated protein kinase (MAPK) profile

Analysis of the phosphorylation states of all MAPKs was performed using a human phospho-MAPK array kit Proteome Profiler™, which is similar to immunoprecipitation and Western blot analysis (R&D Systems, Minneapolis, MN). Human PBMCs were incubated for 60 min with the selected polysaccharide fractions or negative control (HBSS). The cells were rinsed with HBSS and lysed with the buffer provided. Arrays were incubated overnight at 4°C with lysates obtained from 3 × 10^6^ cells for each sample. The arrays were washed three times with 20 ml of the wash buffer provided and incubated for 2 hr with the provided detection antibody cocktail containing phospho-site-specific MAPK biotinylated antibodies. The wash steps were repeated, after which the arrays were exposed to chemiluminescent reagents, and the signal was captured with an Alpha Innotech FluorChem FC2 imaging system.

### EAE induction and treatment

Female 6–8 week old C57BL/6 mice (Frederick Cancer Research Facility, National Cancer Institute, Frederick, MD) were used throughout the study of experimental autoimmune encephalomyelitis (EAE). Mice were maintained under pathogen-free conditions in individual ventilated cages and were fed sterile food and water *ad libitum*. All animal experiments were performed in accordance with National Institutes of Health guidelines and approved by the Montana State University Institutional Animal Care and Use Committee.

For EAE induction, mice were challenged subcutaneously in the flank with 200 μg of myelin oligodendrocyte glycoprotein (MOG)_35–55_ peptide (Biosynthesis, Inc., Lewisville, TX) in 100 μl of incomplete Freund’s adjuvant (IFA) (Sigma–Aldrich, St. Louis, MO) containing 400 μg killed *Mycobacterium tuberculosis* (Difco Laboratories, Detroit, MI) on day 0, as described previously [19]. On days 0 and 2, mice were treated by intraperitoneal (*i.p.*) injection with 200 ng of *Bordetella pertussis* toxin (List Biological Laboratories, Campbell, CA). Starting on day 4, mice were treated daily *i.p.* with 50 mg/kg of **CSP-AU1** in 100 μl of phosphate buffer saline (PBS) or with 100 μl of PBS (control). Mice were monitored and scored daily by a technician blinded to treatments for clinical signs of EAE using the following scoring system: 0, normal; 1, a limp tail; 2, hind limb weakness; 3, hind limb paresis; 4, quadriplegia; 5, moribund state.

### Cytokine analysis in cultures of lymph node (LN) and splenic cells

To assess the cytokines induced, spleen and head and neck LNs were harvested at day 17 of the disease, and total mononuclear cells (5 × 10^6^ cells/ml) were cultured with 10 μg/ml of MOG_35-55_ peptide for 4 days in a complete medium consisting of RPMI 1640 medium with the following supplements (Invitrogen-Life Technologies): 1 mM sodium pyruvate, 1 mM nonessential amino acids, penicillin/streptomycin (10 U/ml), and 10% FBS. TNF, GM-CSF, IL-13, IL-17, IFN-γ, and TGF-β were measured from culture supernatants, as described previously [19].

### Statistical analysis

Mann–Whitney *U* test was used for statistical analysis of clinical scores in the EAE model. One-way ANOVA and Student *t* test were performed to analyze NO and alkaline phosphatase production, MAP kinase phosphorylation, and cytokine-specific ELISA data. Results were considered statistically significant if the *p* value was <0.05. The statistical analysis was performed using Prism 5 (GraphPad Software, San Diego, CA).

## Results

### Ethnopharmacological information

The leaves, roots and stem bark extracts of *C. splendens* (local name: Trokpatan) are used extensively in traditional medicine for treating many diseases in Côte d’Ivoire. To assess the importance of this plant in traditional medicine, 38 healers from the areas of Abidjan, Alépé, Bouaké, Grand-Bassam, Man, M’batto, and Tiassalé in Côte d’Ivoire were interviewed in 2010 and 2012 regarding their use of *C. splendens* (Table 1)*.* Interviewees indicated that *C. splendens* is used for hemorrhoids, sinus disease, menstrual troubles, diarrhea, healing scars, and as a febrifuge and vermifuge agent. The leaves and bark are also used to treat scrofulous infection. Decoction in water (boiling) is the most common mode of preparation. The oral route and the local application are the most commonly used modes of administration. Our ethnopharmacological survey is consistent with many of the previously published uses of *C. splendens* in African folk medicine.

**Table 1:** **Traditional Uses of *Clerodendrum splendens *in Côte d’Ivoire**^**a**^

**Indication**	**Specific parts of the plant**	**Preparation and administration**
Hemorrhoids	Leaves	The leaves were boiled in water, and the decoction was consumed; part of the decoction was mixed in shea butter and applied locally to hemorrhoids
Sinus disease	Leaves	A vapor bath was inhaled once a day for 3 days
Menstrual troubles	Leaves	The leaves were boiled in water, and the decoction was consumed 2 times a day for 2 months
Diarrhea	Leaves	The leaves were boiled in water, and the decoction was consumed 3 times a day for 3 days
Healing scars	Leaves	The pounded fresh leaves were applied as an ointment or rubbed on the scars
Febrifuge agent	Leaves	The leaves were boiled in water, and the decoction was consumed 2 times a day for 1 week
Vermifuge agent	Leaves	The leaves were boiled in water, and the decoction was consumed 2 times a day for 1 week
Scrofulous infection	Leaves and bark	The pounded or crashed fresh leaves and barks were applied or rubbed on skin infections until cured.

^a^Thirty-eight healers were interviewed. No side effects were reported.

### Preparation and partial characterization of *Clerodendrum* polysaccharide fractions

Polysaccharides obtained by ethanol precipitation of *C. splendens* extract were fractionated using DEAE-cellulose, and the resulting fractions, designated as **CSP-N** (***C****.****s****plendens***p**olysaccharide, **n**eutral fraction) and **CSP-A** (***C. s****plendens***p**olysaccharide, **a**cid fraction) were further fractionated using Diaion HP-20 resin to obtain two bound (**CSP-AB** and **CSP-NB**) and two unbound (**CSP-AU** and **CSP-NU**) fractions. The acidic and neutral Diaion-unbound fractions (**CSP-AU** and **CSP-NU**, respectively) were subsequently fractionated by preparative Sepharose 6B size-exclusion chromatography to obtain four sub-fractions, which were selected based on the total carbohydrate and polyphenol elution profiles (designated as **CSP-AU1**, **CSP-AU2**, **CSP-NU1**, and **CSP-NU2**) (Table 2).

**Table 2:** **Biochemical properties of *C. splendens *polysaccharide fractions**

**Fractionation step & Fraction name**			**M.W. (kDa)**	**Type II Arabino-galactan**	**Polyphenol content (%)**	**Methoxyl content (Mol %)**	**DM (%)**^**a**^
**DEAE cellulose**	**Diaion HP-20**	**Sepharose 6B**					
**CSP-A**	**CSP-AB**	**-**	13.2	Negative	9.3 ± 0.29	1.0 ± 0.04	81
	**CSP-AU**	**-**	24.5	Negative	8.8 ± 0.25	0.9 ± 0.04	16
		**CSP-AU1**	38.5	Positive	7.6 ± 0.22	0.9 ± 0.04	30
		**CSP-AU2**	8.6	Negative	8.1 ± 0.14	1.3 ± 0.03	N.D.
**CSP-N**	**CSP-NB**	**-**	12.0	Negative	0.9 ± 0.03	1.1 ± 0.01	67
	**CSP-NU**	**-**	21.7	Positive	0.5 ± 0.01	1.0 ± 0.02	32
		**CSP-NU1**	60.6	Positive	0.2 ± 0.02	N.D.	N.D.
		**CSP-NU2**	5.5	Negative	6.5 ± 0.14	N.D.	N.D.

^a^The degree of methyl esterification (DM) was calculated as the ratio of the molar amount of methanol to the molar amount of uronic acids (galacturonic and glucuronic acids) and is expressed as %. *N.D.* not determined.

Analytical HP-SEC of the individual fractions showed that each fraction was represented by a single and generally symmetrical peak on the chromatogram. Based on calibration curves derived from analysis of pullulan standards, we determined average molecular weights of the polysaccharide fractions. Sequential fractionation of *C. splendens* extract by DEAE-cellulose and Diaion HP-20 gave parent fractions with relatively low molecular weights ranging from 12.0 kDa (fraction **CSP-NB**) to 24.5 kDa (fraction **CSP-AU**). Polysaccharides that bound to the Diaion resin had lower molecular weights in comparison with unbound polysaccharides (13.2 *vs.* 24.5 kDa, and 12.0 *vs.* 21.7 kDa, respectively). Sub-fractions **CSP-AU1** and **CSP-NU1**, which were obtained by size-exclusion chromatography on Sepharose 6B, had relatively high molecular weights of 38.5 and 60.6 kDa, respectively (Table 2). The samples were also tested for possible endotoxin contamination using the LAL assay. All fractions contained less than 0.2 ng endotoxin/mg of polysaccharide, which is considered to be insignificant for various bioactive products [20,21].

Analysis of polyphenols showed that polyphenol content was minimal (<1%) in neutral polysacchride fractions from *C. splendens* (**CSP-NB**, **CSP-NU**, and **CSP-NU1**), whereas all acidic fractions and the low-molecular weight neutral sub-fraction **CSP-NU2** contained a relatively high polyphenol content (from 6.5 to 9.3%). Thus, the polyphenol component of **CSP-NU** is enriched in the low-molecular weight sub-fraction **CSP-NU2**, as compared to the high-molecular weight sub-fraction **CSP-NU1** (Table 2).

Analysis of the polysaccharide fractions using the Yariv test showed that Diaion-unbound fraction **CSP-NU** and sub-fractions **CSP-AU1** and **CSP-NU1** contained type II arabinogalactan, whereas fractions that bound to this resin (**CSP-AB** and **CSP-NB**), unbound fraction **CSP-AU**, and low molecular weight sub-fractions **CSP-AU2** and **CSP-NU2** all tested negative for the presence of type II arabinogalactan (Table 2). Thus, it appears that the relative amount of type II arabinogalactan in parent fraction **CSP-AU** was too dilute to be detected by the Yariv reagent and that it became concentrated enough to be detected in the high-molecular weight sub-fraction **CSP-AU1** after chromatography.

Sugar composition analysis revealed that the polysaccharides from *C. splendens* consisted primarily of galacturonic and glucuronic acids, arabinose, galactose, rhamnose, glucose, and mannose residues (Table 3). Small (<2%) amounts of fucose, xylose, and glucosamine residues were also detected in the fractions. In all fractions studied, arabinose and galactose were the major monosaccharides found, supporting that arabinogalactans are the primary macromolecules. Monosaccharide content in fractions bound and unbound to Diaion HP-20 resin differed by several fold for galacturonic acid, glucose, mannose, and glucosamine residues (Table 3). Analysis of the content of methoxyl groups in the fractions showed that both Diaion-bound fractions **CSP-AB** and **CSP-NB** had a high degree of methyl esterification of the uronic acid residues when compared to the Diaion-unbound fractions (Table 2).

**Table 3:** **Sugar composition of polysaccharide fractions isolated from *C. splendens***^**a**^

**Monosaccharide**	**CSP-AU**	**CSP-AU1**	**CSP-AB**	**CSP-NU**	**CSP-NU1**	**CSP-NB**
	**Mol %**					
Galacturonic Acid	19.6	11.0	2.8	12.3	8.8	6.3
Arabinose	18.1	17.1	22.6	18.1	17.3	23.7
Galactose	17.1	27.0	19.1	31.7	37.7	26.5
Rhamnose	14.0	11.1	15.5	3.1	3.2	4.1
Glucose	9.6	11.1	15.7	5.0	4.8	15.0
Glucuronic Acid	3.9	3.8	3.7	1.6	1.6	1.2
Mannose	2.9	5.6	13.5	7.6	5.2	9.5
Fucose	1.8	1.6	1.7	1.0	1.3	0.9
Xylose	0.6	1.2	0.7	2.0	1.3	1.3
Glucosamine	1.3	1.3	3.5	0.5	0	0.7
Lyxose	0	0	0	0	0	0

^a^Sugar analysis for fractions **CSP-AU2** and **CSP**-**NU2** was not performed.

The acidic fractions were enriched with polyphenols (up 9.5%), whereas the neutral, relatively high molecular weight fractions (**CSP-NB**, **CSP-NU**, and **CSP-NU1**) had minimal amounts of polyphenols (<1%).

### Effects of the *Clerodendrum* polysaccharide fractions on macrophage NO production

**Figure 1:**
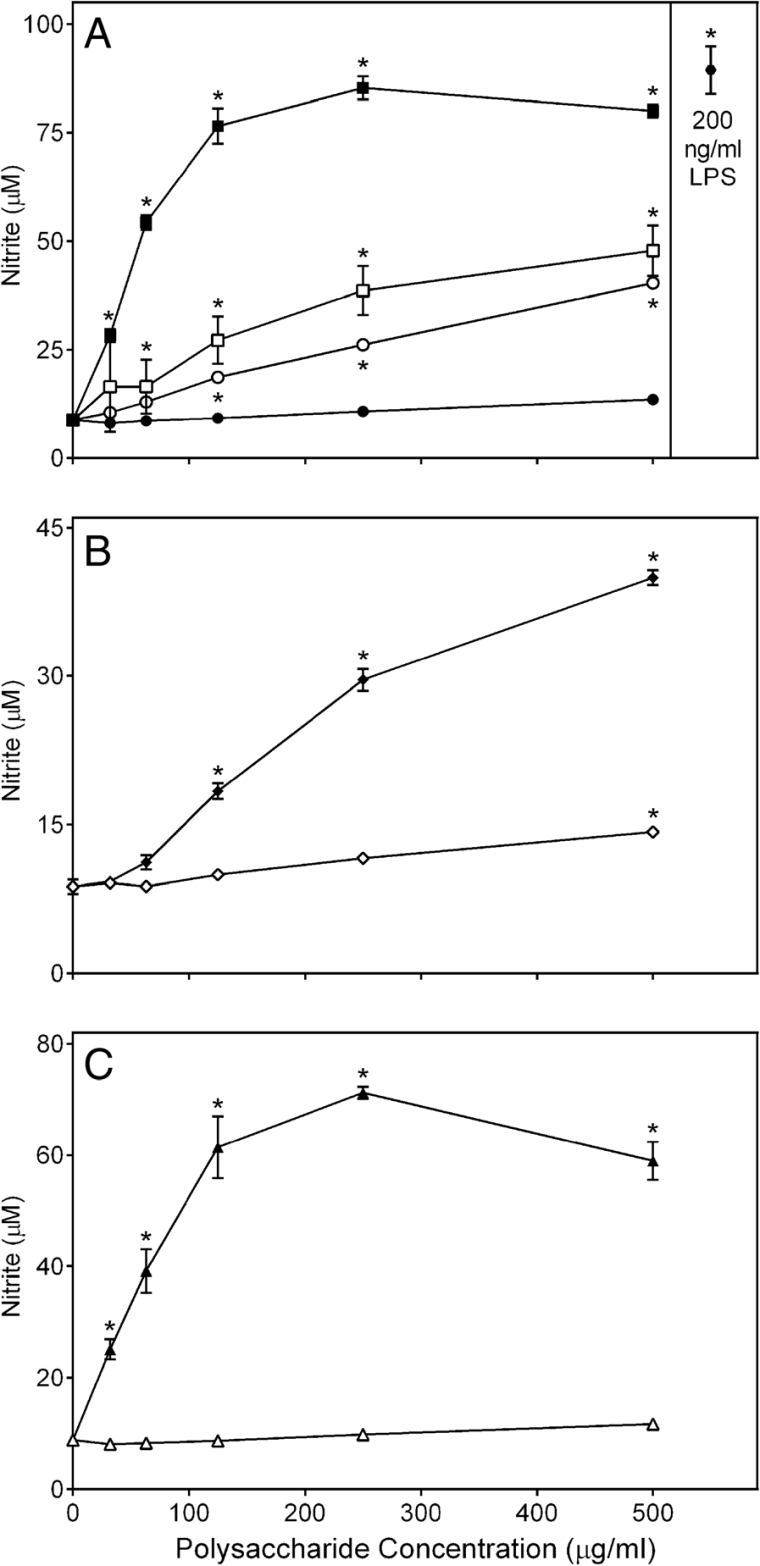
**Effect of *Clerodendrum *polysaccharide fractions on murine macrophage nitric oxide production.** Murine J774.A1 macrophages were incubated for 24 hr with the indicated concentrations of **CSP-NU** (■, panel **A**), **CSP-AU** (□, panel **A**), **CSP-NB** (●, panel **A**), **CSP-AB** (○, panel **A**), **CSP-AU1** (♦, panel **B**), **CSP-AU2** (◊, panel **B**), **CSP-NU1** (▲, panel **C**), **CSP-NU2** (Δ, panel **C**), or 200 ng/ml LPS (, panel **A**). Values are the mean ± S.D. of triplicate samples from one experiment, which is representative of two independent experiments. Statistically significant differences (* p<0.05) between PBS-treated cells and cells treated with polysaccharide fractions or LPS are indicated.

To characterize effect of *C. splendens* polysaccharide fractions in a macrophage cell model, we first evaluated their effects on NO production by murine J774.A1 macrophages. The Diaion-bound neutral fraction **CSP-NB** and low-molecular weight sub-fractions **CSP-AU2** and **CSP-NU2** were inactive or had low activity for induction of macrophage NO production. In contrast, all other fractions were highly active and stimulated levels of macrophage NO production that were comparable to those induced by LPS (Figure 1).

### Effects of *Clerodendrum* polysaccharide fractions on cytokine production in cell cultures

**Figure 2:**
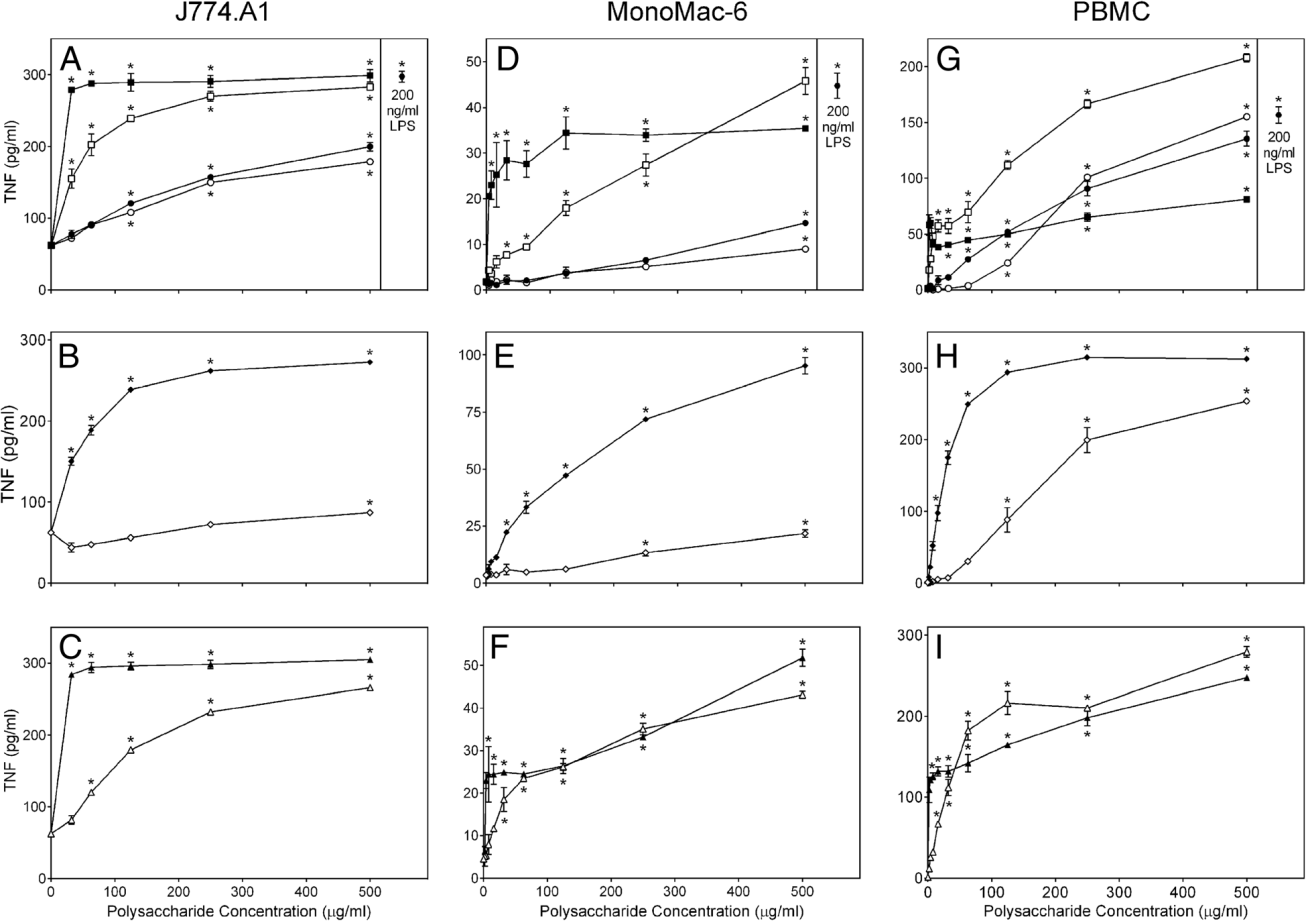
**Effect of *Clerodendrum *polysaccharide fractions on monocyte/macrophage TNF production.** Murine J774.A1 macrophages (panels **A-C**), human MonoMac-6 cells (panels **D-F**), or PBMCs (panels **D-F**) were incubated for 24 hr with the indicated concentrations of CSP-NU (■, panels A, D, and G), CSP-AU (□, panels **A**, **D**, and **G**), CSP-NB (●, panels **A**, **D**, and **G**), CSP-AB (○, panels **A**, **D**, and **G**), CSP-AU1 (♦, panels B, E, and H), CSP-AU2 (◊, panels **B**, **E**, and **H**), CSP-NU1 (▲, panels **C**, **F**, and **I**), CSP-NU2 (Δ, panels **C**, **F**, and **I**), or 200 ng/ml LPS ( , panels **A**, **D**, and **G**). Cell-free supernatants were collected, and extracellular TNF was quantified by ELISA. Values are the mean ± S.D. of triplicate samples from one experiment, which is representative of three independent experiments. Statistically significant differences (* p<0.05) between PBS-treated cells and cells treated with polysaccharide fractions or LPS are indicated.

**Figure 3:**
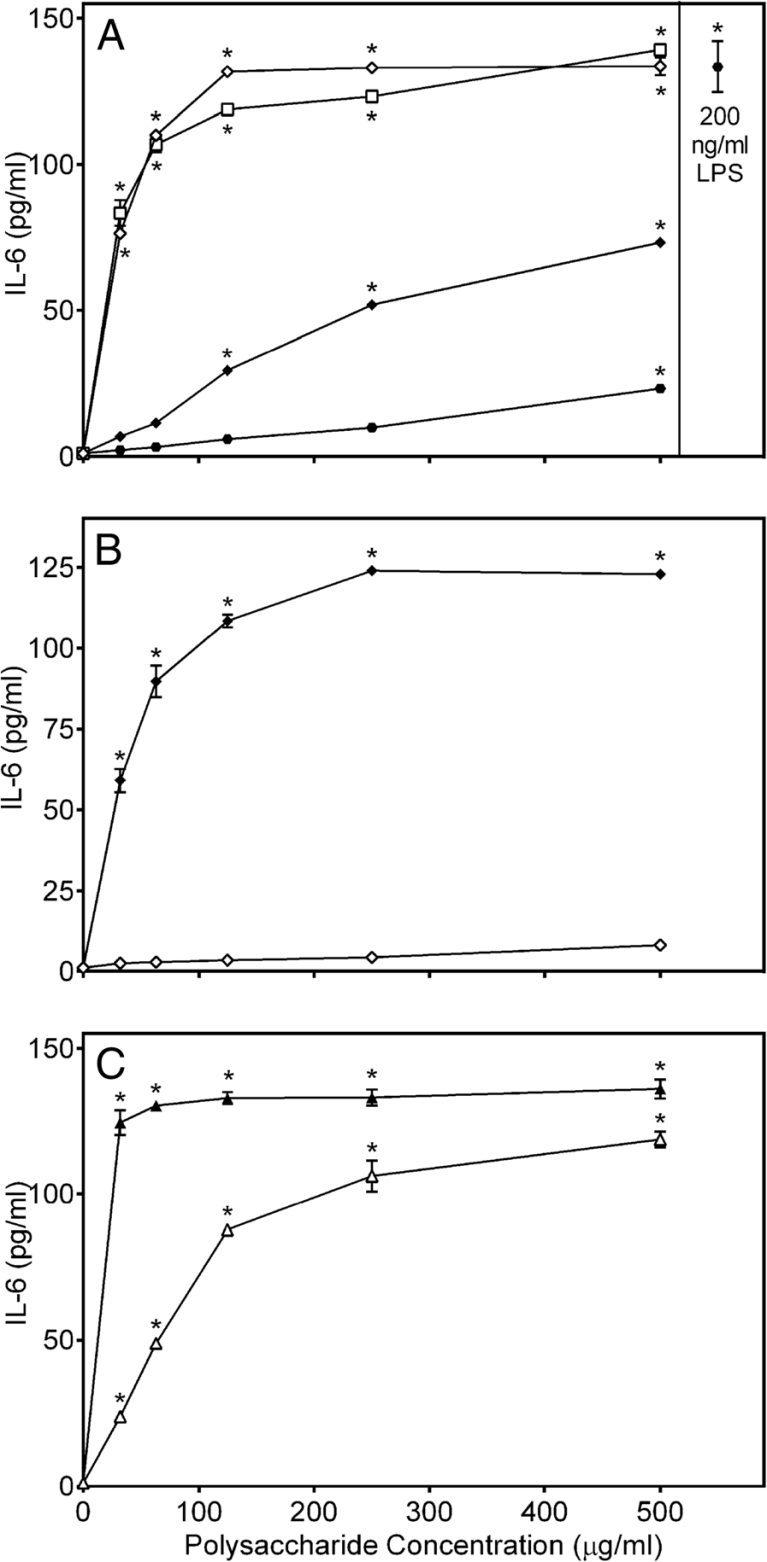
**Effect of *Clerodendrum *polysaccharide fractions on macrophage IL-6 production.** Murine J774.A1 macrophages were incubated for 24 hr with the indicated concentrations of **CSP-NU** (■, panel **A**), **CSP-AU** (□, panel **A**), **CSP-NB** (●, panel **A**), **CSP-AB** (○, panel **A**), **CSP-AU1** (♦, panel **B**), **CSP-AU2** (◊, panel **B**), **CSP-NU1** (▲, panel **C**), **CSP-NU2** (∆, panel **C**), or 200 ng/ml LPS (, panel **A**). Cell-free supernatants were collected, and extracellular IL-6 was quantified by ELISA. Values are the mean ± S.D. of triplicate samples from one experiment, which is representative of three independent experiments. Statistically significant differences (* p<0.05) between PBS-treated cells and cells treated with polysaccharide fractions or LPS are indicated.

The basic mechanism of the immunostimulatory effects of botanical polysaccharides is thought to occur via macrophage stimulation (reviewed in [22]). Thus, we analyzed effects of *C. splendens* polysaccharide fractions on TNF production by murine J774.A1 macrophages, human monocytic MonoMac-6 cells, and human primary PBMCs. We tested samples in murine and human cell lines, as well as primary human cells because we often see differences in macrophage responses between species. Thus, it is important to confirm similar responses in murine phagocytes when considering future *in vivo* studies using a murine model. In addition, confirmation of cell line responses in primary cells is important to confirm cell line responses are also relevant to primary cells. We found that Diaion-bound fractions **CSP-NB** and **CSP-AB** and the acidic low-molecular weight sub-fraction **CSP-AU2** had relatively low activity in J774.A1 and MonoMac-6 cell cultures. All other fractions induced high levels of TNF production by these cell lines, as well as by PBMCs, and the levels of TNF produced were comparable to those induced by LPS (Figure 2). Effects of *Clerodendrum* polysaccharide fractions on IL-6 production were also analyzed in murine J774.A1 macrophages. Again we found that the low-molecular weight sub-fraction **CSP-AU2** and both Diaion-bound fractions **CSP-NB** and **CSP-AB** had low activity, whereas all other *C. splendens* polysaccharide fractions effectively stimulated IL-6 production (Figure 3).

**Figure 4:**
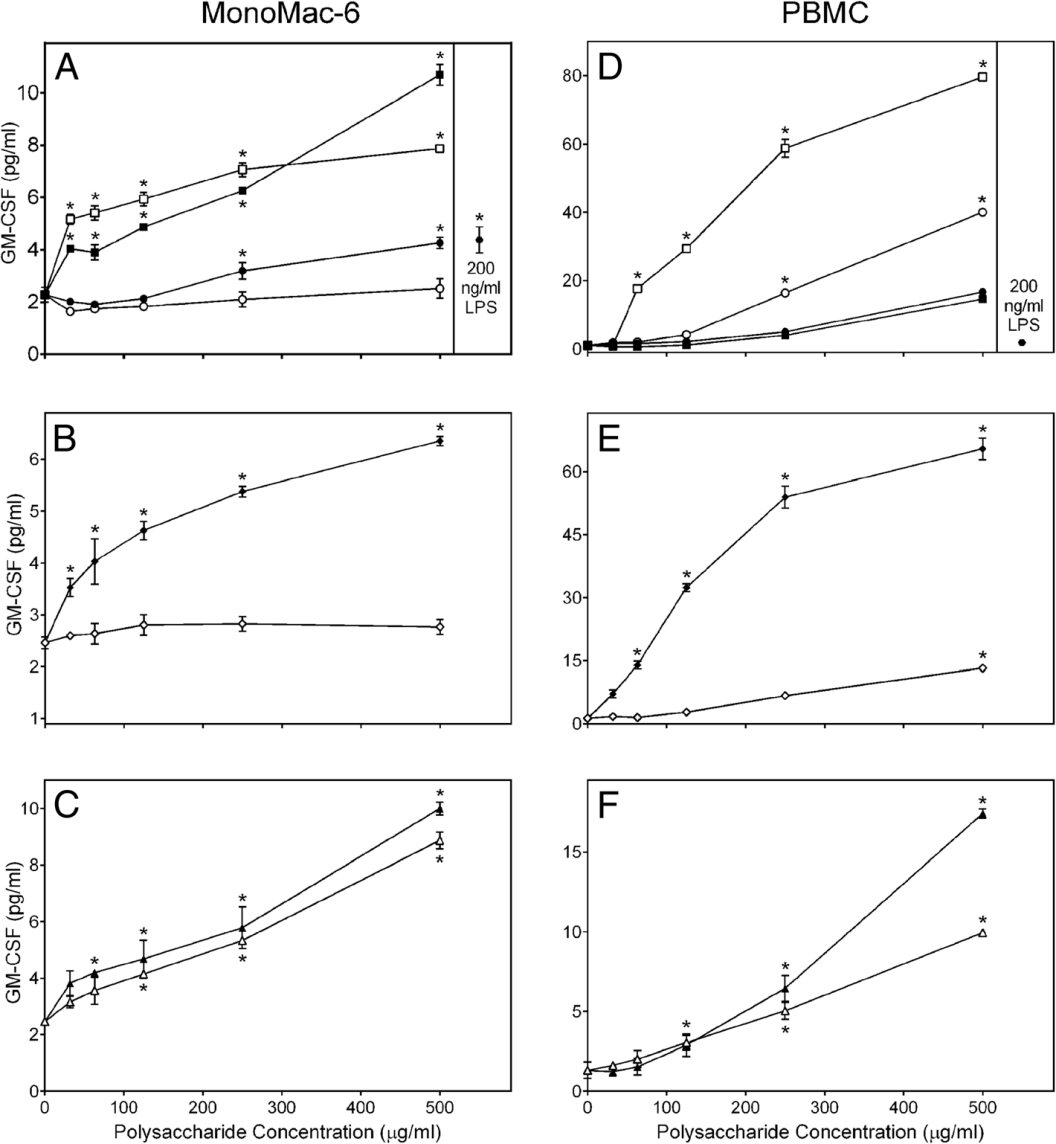
**Effect of *Clerodendrum *polysaccharide fractions on monocyte/macrophage GM-CSF production.** Human MonoMac-6 cells (panels **A-C)** or PBMCs (panels D-F) were incubated for 24 hr with the indicated concentrations of **CSP-NU** (■, panels **A** and **D**), **CSP-AU** (□, panels **A** and **D**), **CSP-NB** (●, panels **A** and **D**), **CSP-AB** (○, panels **A** and **D**), **CSP-AU1** (♦, panels B and E), **CSP-AU2** (◊, panels **B** and **E**), **CSP-NU1** (▲, panels **C** and **F**), **CSP-NU2** (∆, panels **C** and **F**), or 200 ng/ml LPS (, panels **A** and **D**). Cell-free supernatants were collected, and extracellular TNF was quantified by ELISA. Values are the mean ± S.D. of triplicate samples from one experiment, which is representative of three independent experiments. Statistically significant differences (* p<0.05) between PBS-treated cells and cells treated with polysaccharide fractions or LPS are indicated.

**Figure 5:**
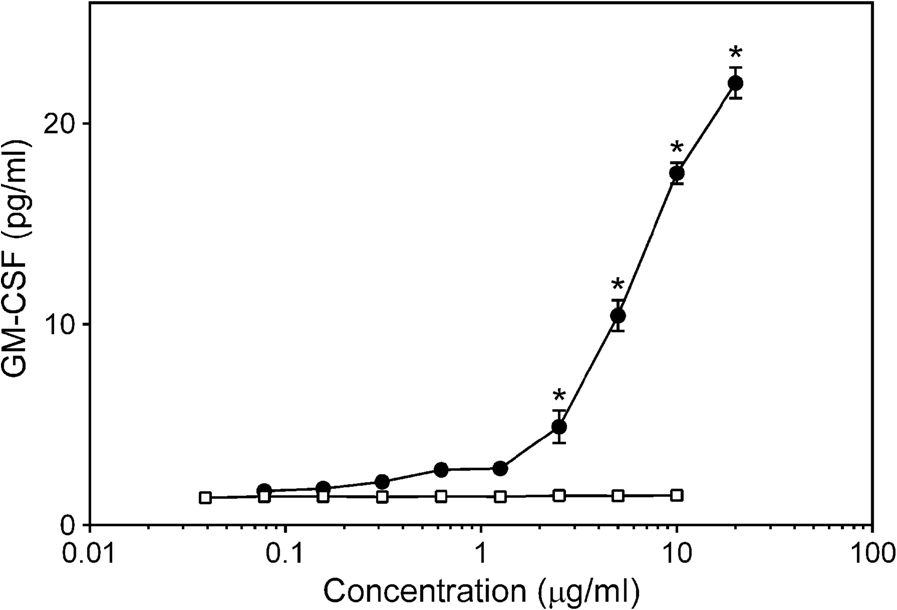
**Effect of Con A and LPS on GM-CSF production by human PBMCs.** PBMCs were incubated for 24 hr with the indicated concentrations of Con A (○) or LPS (□). Cell-free supernatants were collected, and extracellular GM-CSF was quantified by ELISA. Values are the mean ± S.D. of triplicate samples from one experiment, which is representative of two independent experiments. Statistically significant differences (* p<0.05) between PBS-treated cells and cells treated with Con A or LPS are indicated.

Similarly, all *C. splendens* polysaccharide fractions except the Diaion-bound polysaccharide fractions **CSP-NB** and **CSP-AB** and the low-molecular weight sub-fraction **CSP-AU2**, induced high levels of GM-CSF production by MonoMac-6 cells and PBMCs (Figure 4). Note, however, that even high concentrations of LPS (up to 10 μg/ml) were unable to stimulate GM-CSF production by human PBMCs. In contrast, Con A dose-dependently stimulated production of this cytokine by PBMCs over a concentration range of 1 to 20 μg/ml (Figure 5).

To determine if polysaccharides from different plant sources could stimulate similar levels of GM-CSF production, we evaluated the effect of polysaccharide fractions from several different plants. These previously characterized polysaccharide fractions included fraction **A-I** from *Artemisia tripartite*[23], fraction **T-I** from *Tanacetum vulgarie*[24], and fraction **C-I** from *Opuntia polyacanta*[25]. Interestingly, none of these polysaccharide fractions induced GM-CSF production by human PBMCs (data not shown). On the other hand, our previous studies showed that these polysaccharides could potently activate production of various other cytokines, such as TNF and IL-6, in phagocytes [23-25].

**Figure 6:**
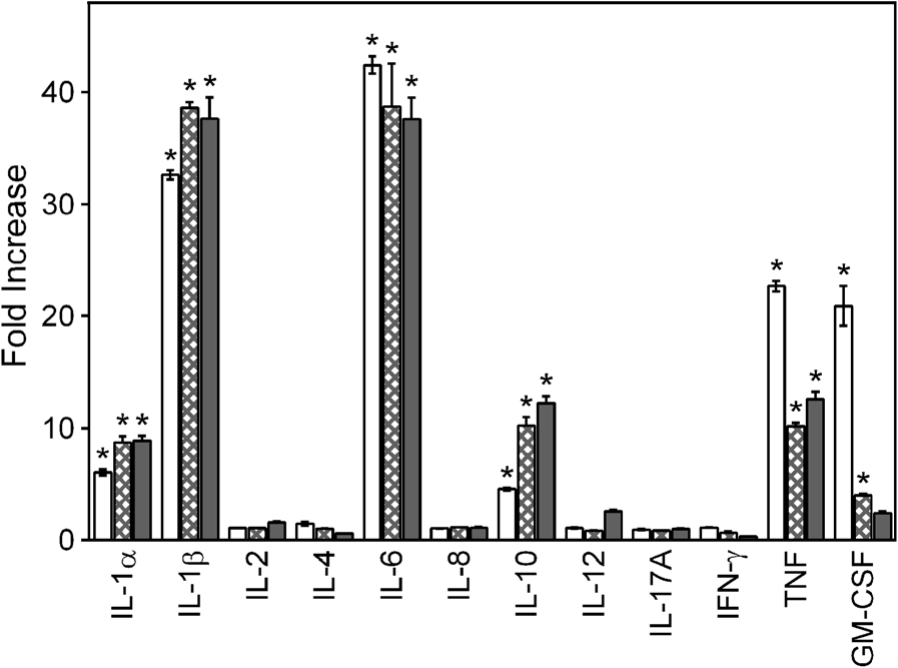
**Cytokine array analysis of PBMC cytokine production after treatment with *Clerodendrum *polysaccharide fractions CSP-AU1 and CSP-NU1.** Human PBMCs were incubated for 24 hr with 250 μg/ml of **CSP-AU1** (open bars) and **CSP-NU1** (hatched bars), or 200 ng/ml of LPS (solid bars), and production of cytokines in the supernatants was evaluated using a MultiAnalyte ELISArray kit. Cytokine expression is shown as fold increase compared to background (PBS-treated cells). The data are presented as mean ± SD of duplicate samples from one experiment that is representative of two independent experiments. Statistically significant differences (* p<0.05) between PBS-treated cells and cells treated with polysaccharide fractions or LPS are indicated.

Because **CSP-AU1** was one of the most potent fractions for activation of TNF, IL-6, and GM-CSF production, we next determined whether this sub-fraction could modulate production of other cytokines by PBMCs using a multi-cytokine ELISA semiquantitative array. Among the 12 cytokines analyzed, six were consistently induced in PBMCs (>4-fold) by 250 μg/ml of **CSP-AU1**, as compared with control (HBSS-treated) cells. These included IL-1α (FI= 6.0), IL-1β (FI = 32.6), IL-6 (FI= 42.4), IL-10 (FI= 4.6), TNF (FI = 22.7), and GM-CSF (FI= 20.9) (Figure 6). IL-8 production was inconclusive because of its high background production by PBMCs (data not shown), a problem which has also been documented previously (e.g., [26]). Thus, these results confirmed activation of IL-6, TNF, and GM-CSF production by **CSP-AU1**, but also that this polysaccharide fraction induced IL-1α, IL-1β, and IL-10 production.

### Effect of the *Clerodendrum* polysaccharide fractions on AP-1/NF-κB activity

**Figure 7:**
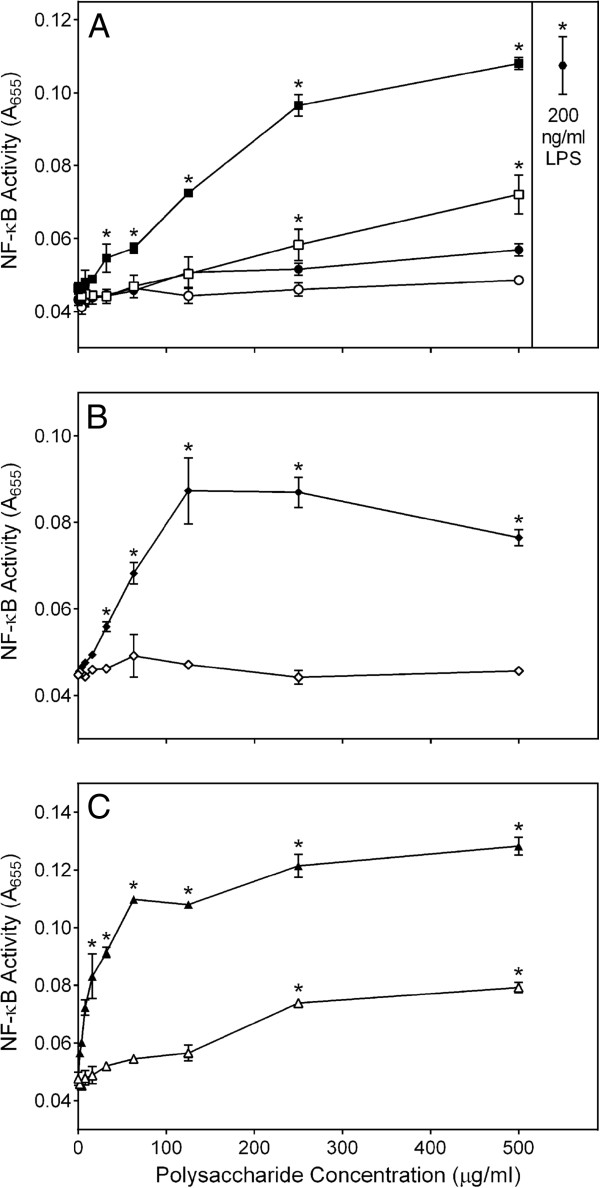
**Effect of *Clerodendrum *polysaccharide fractions on AP-1/NF-κB activation.** Human THP-1Blue monocytes were incubated for 24 hr with the indicated concentrations of **CSP-NU (**■, panel **A)**, **CSP-AU (**□, panel **A)**, **CSP-NB (**●, panel **A)**, **CSP-AB (**○, panel **A)**, **CSP-AU1 (**♦, panel **B)**, **CSP-AU2 (**◊, panel **B)**, **CSP-NU1 (**▲, panel **C)**, **CSP-NU2 (**∆, panel **C)**, or 200 ng/ml LPS (, panel A). Alkaline phosphatase activity was analyzed spectrophotometrically (absorbance at 655 nm) in the cell supernatants, as described. Values are the mean ± S.D. of triplicate samples from one experiment, which is representative of two independent experiments. Statistically significant differences (* p<0.05) between PBS-treated cells and cells treated with polysaccharide fractions or LPS are indicated.

To evaluate signaling pathways involved in the immunomodulatory activity of *C. splendens* polysaccharide fractions, we utilized a transcription factor-based bioassay for AP-1/NF-κB activation in human THP-1Blue monocytes. Similar to activity of the fractions in our analysis of cytokine production, both Diaion-bound fractions **CSP-NB** and **CSP-AB** and the low-molecular weight sub-fraction **CSP-AU2** were inactive or had low activity for inducing AP-1/NF-κB. In contrast, **CSP-NU** and both high-molecular weight sub-fractions **CSP-AU1** and **CSP-NU1** dose-dependently induced AP-1/NF-κB transcriptional activity, as determined by monitoring alkaline phosphatase release (Figure 7).

### Effect of *Clerodendrum* polysaccharide fractions on cell viability

Although our functional assays suggested that the polysaccharides extracted from *C. splendens* were relatively non-toxic, we evaluated their potential cytotoxic effects to determine if the results might be influenced by background toxicity. Using a cytotoxicity assay, we determined that none of the polysaccharides significantly affected viability of human PBMCs, THP-1Blue, or MonoMac-6 cells over the concentration range of 10–250 μg/ml polysaccharides, verifying that these polysaccharides were not toxic to these cells (data not shown).

### Effect of CSP-AU1 on TLR4-dependent pathway and phosphorylation of MAPK family

**Figure 8:**
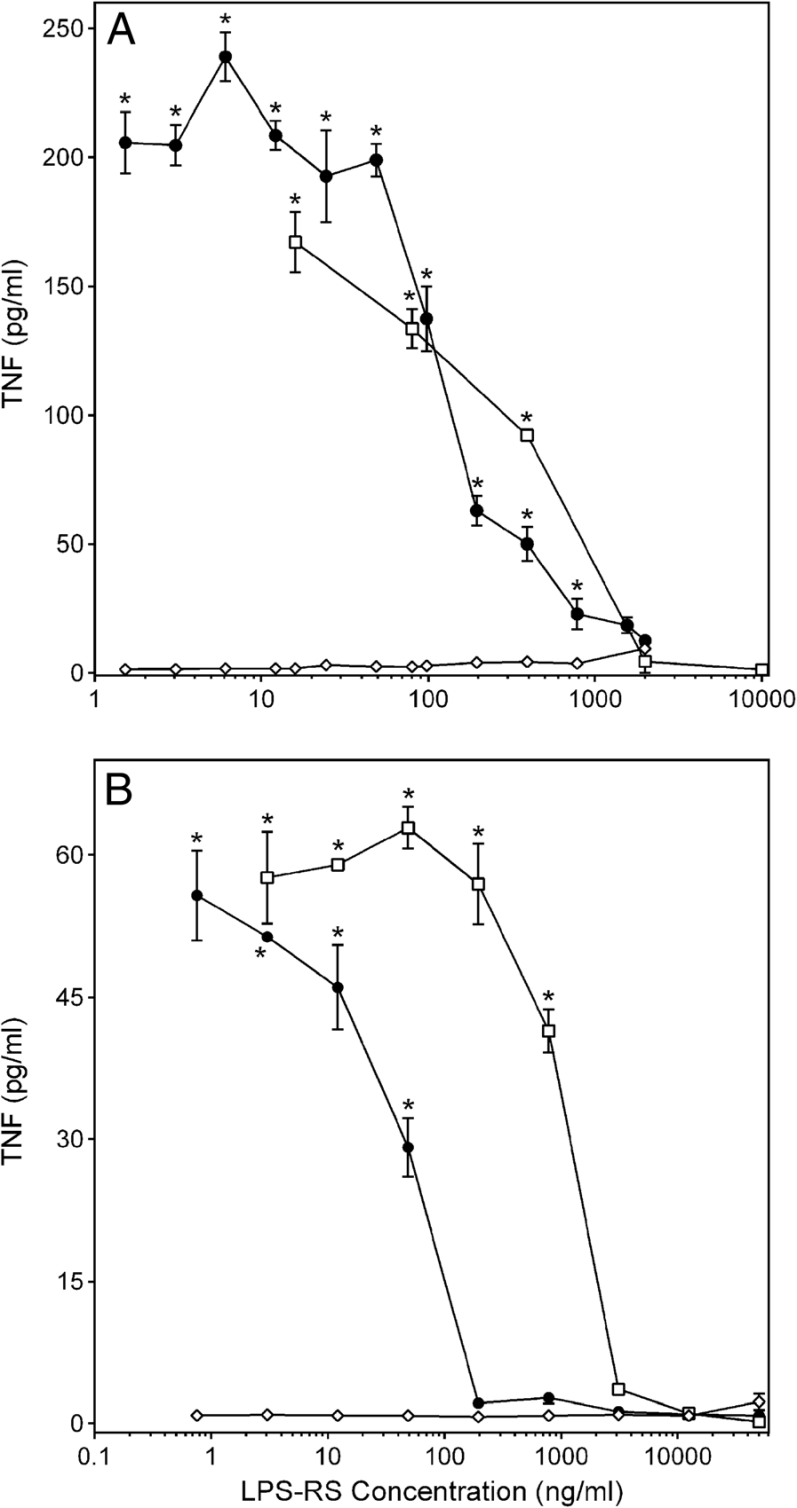
**Effect of TLR4 antagonist LPS-RS on TNF production by monocyte/macrophages activated with *Clerodendrum *polysaccharide fraction CSP-AU1.** Human PBMCs (Panel **A**) or MonoMac-6 cells (Panel **B**) were pretreated with indicated concentrations LPS-RS for 30 min, followed by treatment with media (negative control, ●), **CSP-AU1** (250 μg/ml, ○), or LPS (200 ng/ml, □) for 24 hr, and extracellular TNF was quantified by ELISA. Values are the mean ± S.D. of triplicate samples from one experiment, which is representative of three independent experiments. Statistically significant differences (* p<0.05) between PBS-treated cells and cells treated with the indicated concentrations of LPS-RS plus fraction **CSP-AU1** or LPS are indicated.

LPS from the photosynthetic bacterium *Rhodobacter sphaeroides* (LPS-RS) is a potent TLR4 antagonist, and the primary mechanism of the antagonism consists of direct competition with LPS from *E. coli* or other pathogenic bacteria for the same binding site on myeloid differentiation protein 2 (MD-2), an essential co-receptor in the LPS/TLR4 signaling pathway [27]. As shown in Figure 8, LPS-RS alone had a negligible effect on TNF production in PBMCs and MonoMac-6 cells. However, LPS-RS (2 μg/ml) completely inhibited TNF secretion by PBMCs activated with 250 μg/ml of **CSP-AU1** or 200 ng/ml of LPS (Figure 8A). The effect of this TLR4 antagonist on TNF secretion by MonoMac-6 cells was fairly similar; 10 and 0.2 μg/ml of LPS-RS completely inhibited TNF secretion by MonoMac-6 cells induced by 250 μg/ml of **CSP-AU1** and 200 ng/ml of LPS, respectively (Figure 8B). It should be noted that LPS-RS was unable to inhibit TNF secretion by PBMCs activated with 200 nM PMA (data not shown).

**Figure 9:**
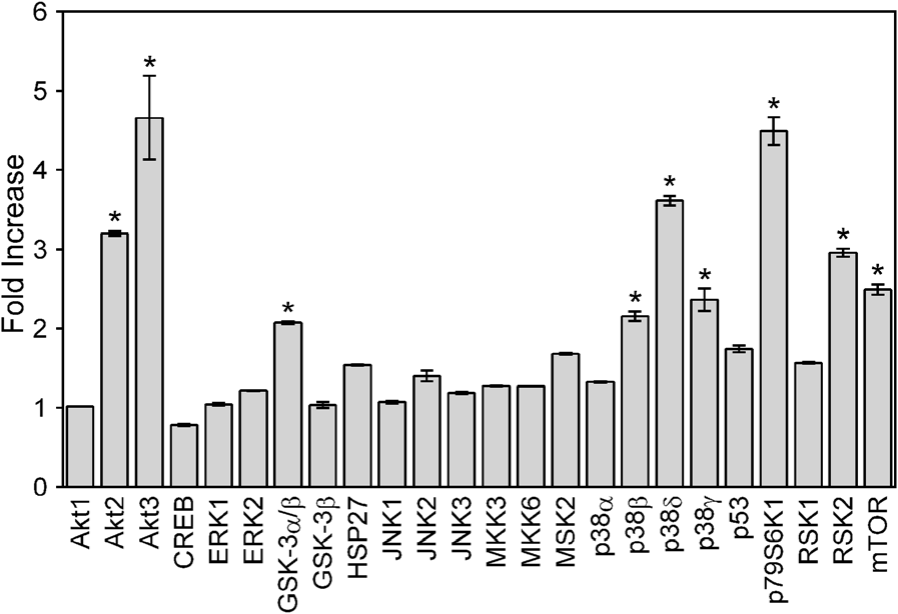
**Effect of CSP-AU1 on MAP kinase phosphorylation.** PBMCs were incubated for 30 min with 250 μg/ml of **CSP-AU1**, and levels of kinase phosphorylation were evaluated using a phospho-MAPK array kit. The data are presented as mean ± SD of duplicate samples from one experiment. Statistically significant differences (* p<0.05) between PBS-treated cells and cells treated with **CSP-AU1** are indicated.

Stimulation of cytokine production by monocyte/macrophages depends on multi-signaling pathways, including MAPKs [28]. To simultaneously evaluate effects of fraction **AP-AU1** on the activation status of all three major MAPKs, extracellular-signal regulated kinases (ERK1/ERK2), c-Jun N-terminal kinases (JNK 1–3), different p38 MAPK isoforms (α, β, δ, and γ) and other intracellular kinases, such as MSK2, mTOR, CREB, HSP27, p53, Akt, glycogen synthase kinase (GSK-3), p90 ribosomal S6 kinase (RSK)1/2, MAP kinase kinases (MKK2, MKK3, and MKK6), and p70 S6 kinase 1 (p70S6K1) in hPBMCs, we utilized a human phospho-MAPK array (Proteome Profiler™; R&D Systems), which monitors the phosphorylation of multiple intracellular kinases. Treatment with **CSP-AU1** specifically increased (>2-fold) phosphorylation Akt2 [Ser^474^; fold increase (FI)= 3.2], Akt3 (Ser^472^, FI=4.7), GSK-3α/β (FI=2.1), p38β (Thr^221^/Tyr^223^, FI= 2.2), p38δ (Thr^180/Tyr182^; FI= 3.6), p38γ (Thr^183^/Tyr^185^; FI= 2.4), p70S6K1 (Thr^421^/Ser^424^; FI= 4.5), RSK2 (Ser^386^; FI= 3.0), and mTOR (Ser^2448^; FI= 2.5) in human PBMCs (Figure 9).

### Effect of CSP-AU1 on cytokine concentration in serum

**Figure 10:**
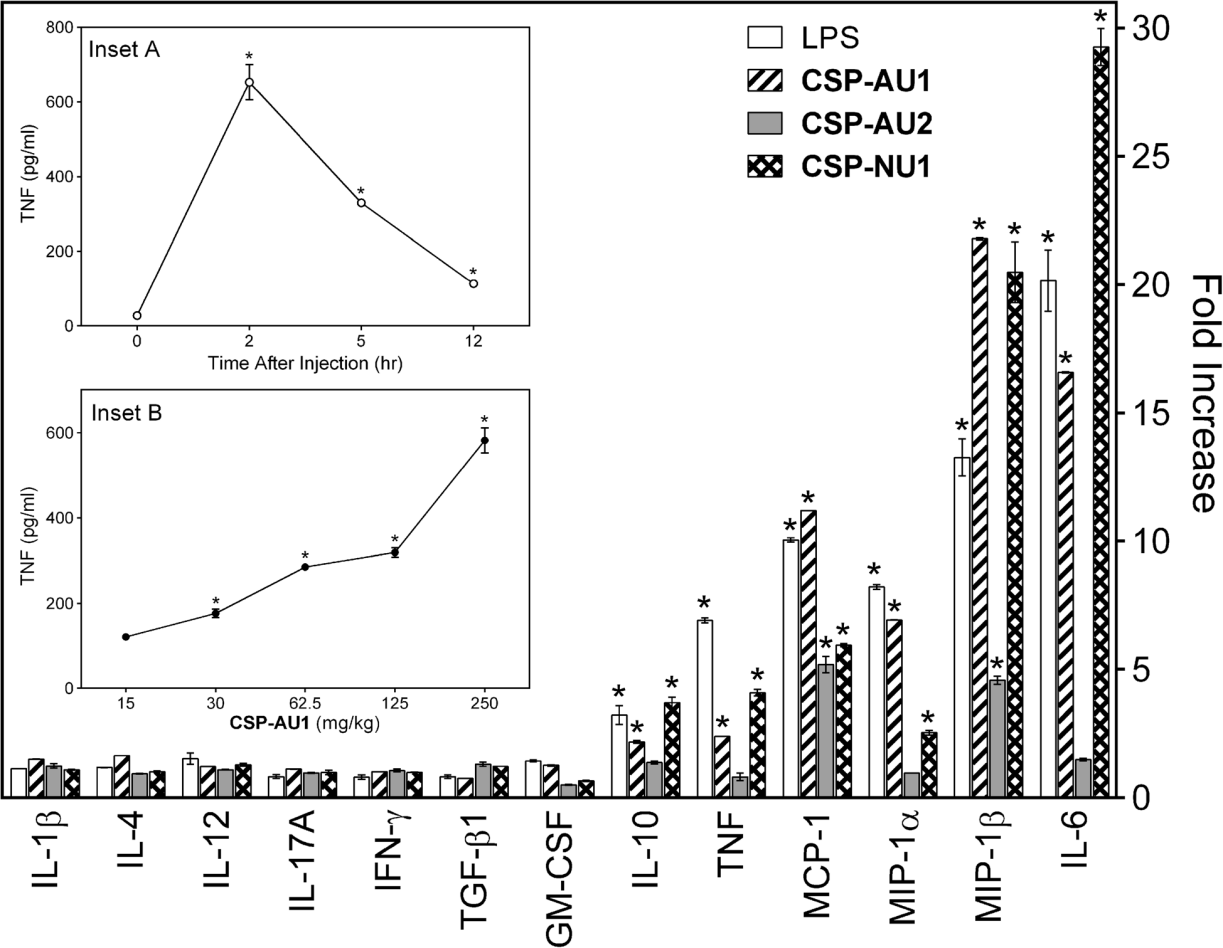
**Effect of *Clerodendrum *polysaccharide fractions on serum cytokines.** Balb/c mice (n=4) were treated by *i.p*. injection with polysaccharide fractions **CSP-AU1**, **CSP-AU2**, **CSP-NU1** (single dose of 250 mg/kg in 100 μl of PBS), LPS (single dose of 500 μg/kg in 100 μl of PBS), or PBS (100 μl). After 2 hr, mice were sacrificed, and serum was collected. Serum cytokine levels were evaluated using a MultiAnalyte ELISArray kit. Cytokine levels are shown as fold increase compared to background (PBS-treated mice). ***Inset A***. **CSP-AU1** was administrated *i.p*. (single dose 250 mg/kg), mice (n=3) were sacrificed at the indicated time points, and serum was collected. ***Inset B***. The indicated doses of **CSP-AU1** were administrated *i.p.*, the mice (n=3) were sacrificed after 2 hr, and serum was collected. Serum TNF levels were evaluated using ELISA. For the Figure and Insets, the data are presented as mean ± S.D. of triplicate samples from different mice and are representative of two independent experiments. Statistically significant differences (* p<0.05) between animals treated with PBS and **CSP-AU1** or LPS are indicated.

Both high activity (**CSP-AU1** and **CSP-NU1**) and low activity (**CSP-AU2**) fractions were evaluated for their ability to modulate production of different cytokines in serum using a multi-cytokine semiquantitative ELISA array. We found that the profiles of cytokine-stimulatory activity of **CSP-AU1** and **CSP-NU1** were comparable to that of LPS (single dose of 500 μg/kg). Among the 13 cytokines analyzed, six were consistently increased in serum (>2-fold) by a single dose of **CSP-AU1** or **CSP-NU1** (250 mg/kg) at 2 hr, as compared with control (PBS-treated) mice. These included IL-10 (FI= 2.2 and 3.7), IL-6 (FI= 16.6 and 29.3), TNF (FI = 2.4 and 4.1), monocyte chemoattractant protein-1 (MCP-1) (FI= 11.2 and 6.0), MIP-1α/CCL3 (FI= 6.9 and 2.5), and MIP-1β/CCL4 (FI = 21.8 and 20.5, by sub-fractions **CSP-AU1** and **CSP-NU1**, respectively) (Figure 10). It should be noted that only two cytokines MCP-1 (FI= 5.2) and MIP-1β/CCL4 (FI= 4.6) were significantly increased in serum (>2-fold) by injection of the low molecular weight sub-fraction **CSP-AU2**, which correlated with its low activity in our *in vitro* assays.

Because **CSP-AU1** was the most abundant fraction among the isolated *Clerodendrum* polysaccharide fractions (its yield was ~5 times larger than yield of **CSP-NU1**; data not shown), we used **CSP-AU1** for subsequent *in vivo* studies. After *i.p.* administration of **CSP-AU1** (single injection in dose of 250 mg/kg), a rapid rise in serum TNF concentration was observed, peaking at ~2 hr (Figure 10, *Inset A*). When mice were treated with different doses of **CSP-AU1**, a dose–dependent increase in serum TNF concentration was found at 2 hr (Figure 10, *Inset B*).

### Effect of CSP-AU1 on EAE

**Figure 11:**
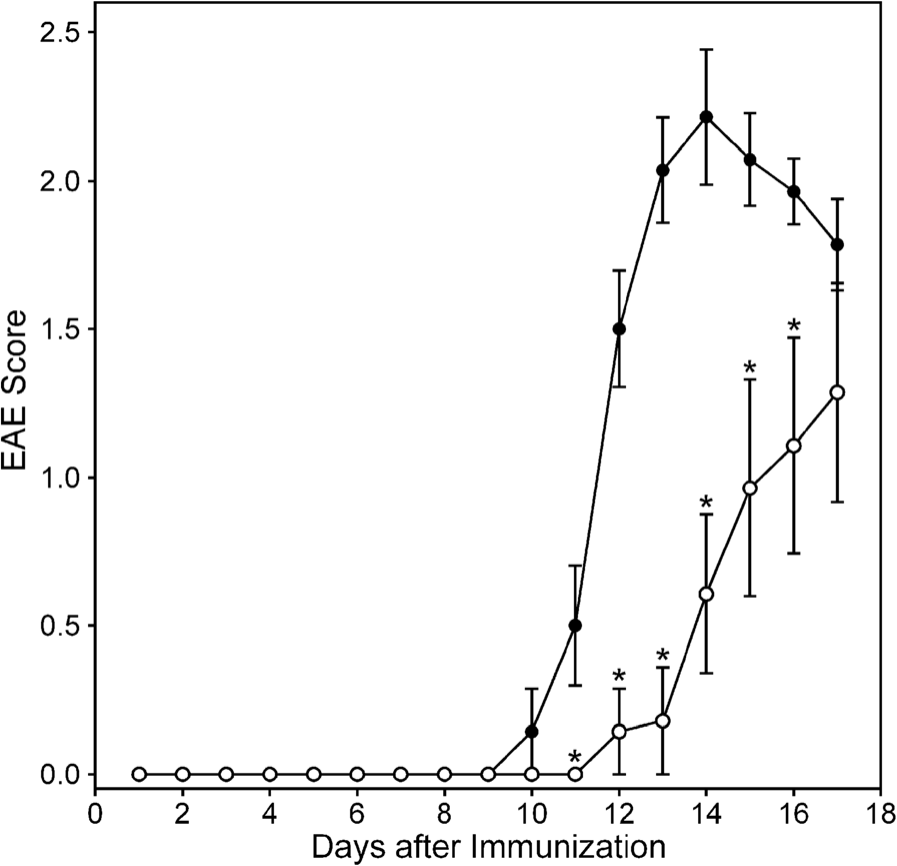
**Effect of CSP-AU1 on EAE.** C57BL/6 mice were treated *i.p.* daily with 50 mg/kg of **CSP-AU1** (○) or PBS (●), starting on day 4 after immunization with MOG_35-55_ peptide. The average score of 14 mice per group is shown, and statistically significant differences (* p<0.05) between EAE mice treated with PBS or fraction **CSP-AU1** are indicated. A representative experiment of two independent experiments is shown.

Because **CSP-AU1** had potent TLR4-dependent immunomodulatory effects via MD-2, we hypothesized that this polysaccharide might have a clinically beneficial effect on autoimmune diseases. Indeed, chronic stimulation of the innate immune system via TLR4 leads to the induction of immunosuppression [29], and several plant polysaccharide with immunomodulatory activities were recently found to have therapeutic effects in several autoimmune disease models, including EAE [30-34]. In multiple sclerosis and its animal model EAE, myelin auto-reactive T cells and macrophages migrate into the central nervous system via a broken blood–brain barrier. The inflammatory cascade stimulated by these cells ultimately leads to neuroinflammatory injury and myelin sheath destruction [35]. To test whether **CSP-AU1** could down-regulate inflammation in EAE, mice were treated daily (starting on day 4 after immunization with MOG_35-55_ peptide) with 50 mg/kg of **CSP-AU1**. Notably, we found that **CSP-AU1** effectively delayed onset of EAE by 2 days as well as decreasing disease severity. In the PBS-treated group, EAE developed at day 10 after challenge and reached a maximal clinical score at day 14. In contrast, the polysaccharide-treated group had a delayed onset of symptoms (day 11) and far less severe disease, reaching a maximal clinical score at day 17 (insignificant difference with PBS-treated group at day 17, p>0.05) (Figure 11).

**Figure 12:**
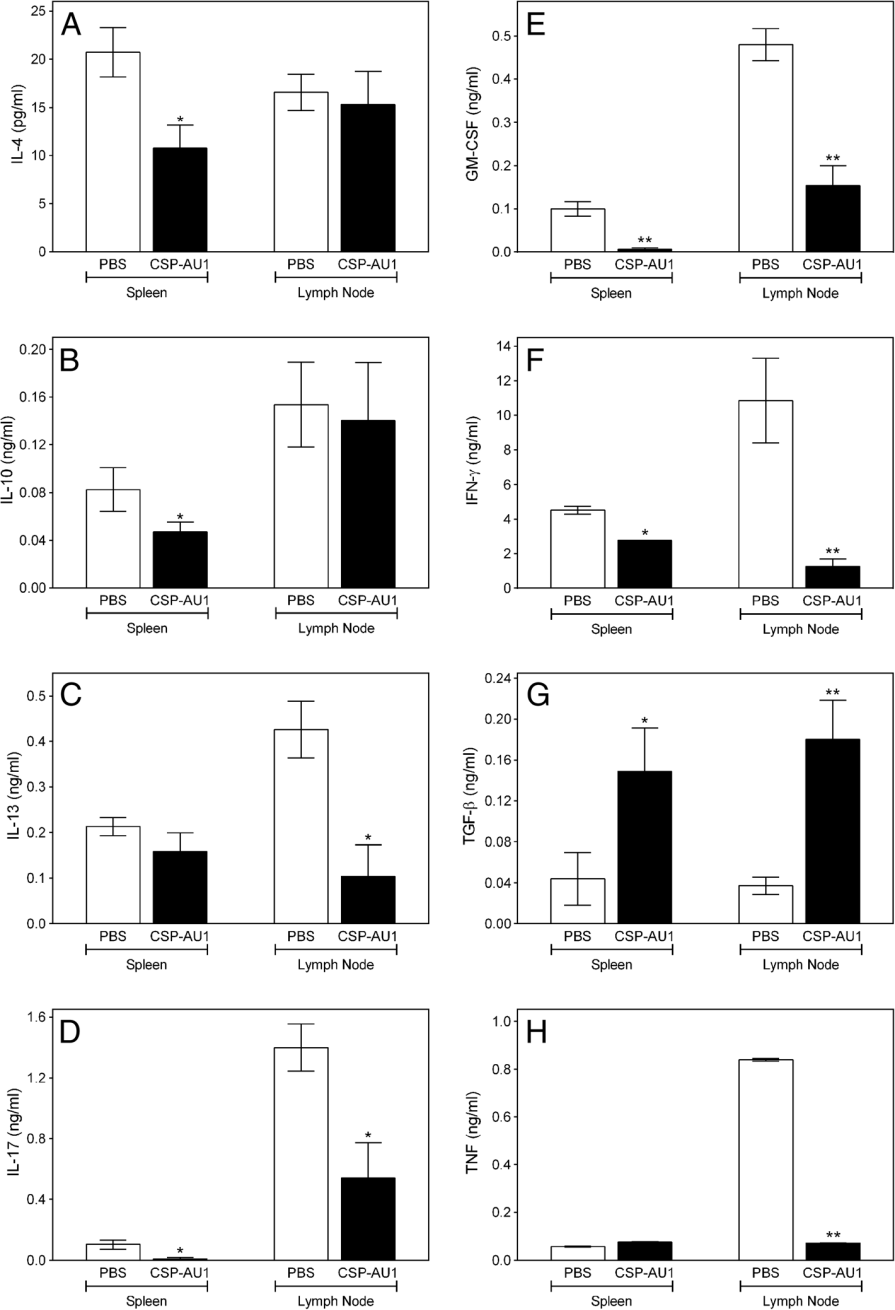
**Cytokine profile of splenic and lymph node (LN) cells isolated from PBS- or CSP AU1-treated EAE mice.** Splenic and LN cells were prepared from mice at day 17 after immunization with MOG_35-55_ peptide. These cells (5 × 10^6^ cell/ml) were restimulated with 10 μg/ml MOG_35-55_ for 4 days, cell-free supernatants were collected, and extracellular cytokines were quantified by ELISA. Mean cytokine concentrations from 3–5 cultures ± S.D. are shown. Statistically significant differences (* p<0.05; ** p<0.001) between cells, isolated from PBS- and **CSP-AU1**-treated EAE mice are indicated.

To understand the mechanisms responsible for reduced EAE disease in **CSP-AU1**-treated mice, mononuclear cells from spleens and head and neck LNs were isolated from **CSP-AU1**- and PBS-treated EAE mice on day 17 after immunization and cultured 4 days with the MOG_35-55_ peptide. As shown in Figure 12, high levels of IL-13, IL-17, GM-CSF, INF-γ, and TNF were produced in LN cells from PBS-treated EAE mice, while secretion of all of these cytokines was significantly decreased in cultures of these cells from **CSP-AU1**-treated EAE mice. The levels of anti-inflammatory cytokines IL-4 and IL-10 were similar in the LN supernatants from **CSP-AU1**-treated and control animals. For splenic cells, decreased IL-4, IL-10, IL-17, GM-CSF, and IFN-γ levels were found in **CSP-AU1**-treated mice. On the other hand, TGF-β was increased in both splenocytes and LN cells from **CSP-AU1**-treated EAE mice relative to PBS control mice (Figure 12).

## Discussion

Historically, *C. splendens* has been used in African folk medicine for treating a number of diseases and medical conditions. However, despite the widespread traditional use of *C. splendens* in tropical countries, little is known regarding the active components responsible for its therapeutic properties. Previously, antipyretic and anti-inflammatory effects of methanolic extracts from *C. splendens* have been described [11], whereas the immunomodulatory activities of high-molecular weight polysaccharide fractions from this plant are unknown. In the present work, we provide evidence demonstrating that *C. splendens* polysaccharides have potent immunomodulatory properties *in vitro* and *in vivo* and suggest that this may contribute, at least in part, to the therapeutic potential of *C. splendens*-derived medicines.

Our ethnobotanical survey and literature review indicate that a decoction in water of the leaves is the mode of extraction most commonly used to prepare remedies from *C. splendens* in African folk medicine. Based on this observation, we isolated eight polysaccharide fractions from the hot-water extract of *C. splendens* leaves and provided initial structural and pharmacological characterization. Average molecular weights of the fractions were in the range of 5.5 to 60.6 kDa. We found that the high-molecular weight sub-fractions **CSP-AU1** (mol. weight 38.5 kDa) and **CSP-NU1** (mol. weight 60.6 kDa) were quite potent in biological tests, whereas the low-molecular weight sub-fraction **CSP-AU2** was inactive or minimally active in the same assays. Indeed, sub-fractions **CSP-AU1** and **CSP-NU1** contained potent immunomodulatory activity, as demonstrated by their ability to induce NO production in macrophage J774.A1 cells and cytokines by monocyte/macrophages and human PBMCs. These cytokines included of IL-1α, IL-1β, IL-6, TNF, IL-10, and GM-CSF. In addition, we found that **CSP-AU1** and **CSP-NU1** induced increased levels of certain serum cytokines *in vivo*, including the anti-inflammatory cytokine IL-10 and pro-inflammatory cytokines IL-6, TNF, MCP-1, MIP-1α, and MIP-1β. In contrast, the low-molecular weight sub-fraction **CSP-AU2** was minimally active *in vivo* (Figure 10).

A common feature of plant polysaccharides that modulate macrophage functions may be higher molecular weight, as we found previously that immunomodulatory activity positively correlated with increased molecular weight of various plant polysaccharides [23-25,36]. Although the immunologic effects of *Clerodendrum* polysaccharides following oral administration were not studied in the present work, numerous dietary polysaccharides do appear to elicit diverse immunomodulatory effects in various animal tissues, including the blood, gastrointestinal tract and spleen [37-42]. Note, however, that our recent studies suggest that nasal application of some plant polysaccharides may be even more effective than oral administration for stimulation of innate immunity [43]. In any case, further studies are now in progress to determine the therapeutic potential of *Clerodendrum* polysaccharide sub-fraction **CSP-AU1** and its structural features conferring biological activity.

Active and inactive polysaccharide fractions contained polyphenols, which were present over a broad range. Polyphenols have been previously noted in polysaccharide fractions of various plants (e.g. [42,44]), and it has been reported that polysaccharides are able to bind to polyphenols by intermolecular interactions [45], changing the molecular conformation of carbohydrates. Therefore, the presence of polyphenols in polysaccharide fractions must be considered. However, activities of the fractions did not correlate with polyphenol content in our biological studies. Indeed, one of the most active fractions (**CSP-AU1**) contained a relatively high level of polyphenols (7.6%), whereas other polyphenol-enriched fractions (**CSP-AB**, 9.3% polyphenols; **CSP-AU2**, 8.8% polyphenols) were inactive or had low activity in most biological assays. Finally, the highly active sub-fraction **CSP-NU1** had the lowest polyphenol content (0.2%).

We found that both of the Diaion-bound fractions, **CSP-AB** and **CSP-NB**, generally were inactive or had low biological activity. Sugar analysis showed that these fractions contained much less galacturonic acid but more neutral sugars (glucose and mannose) than the Diaion-unbound fractions **CSP-AU** and **CSP-AU**. Moreover, analysis of the fractions using the Yariv reagent demonstrated that both **CSP-AB** and **CSP-NB** tested negative for the presence of type II arabinogalactan. Conversely, the most potent immunomodulatory properties were associated with the fractions that exhibited a positive reaction for type II arabinogalactan. Thus, native type II arabinogalactans could be the main structures responsible for immunomodulatory properties of the *C. splendens-*derived polysaccharides, although presence of type I arabinogalactan in the fractions could not be excluded. In any case, it appears that Diaion HP-20 resin could be used to enrich bioactive *C. splendens* polysaccharides by removing inactive material that bound to the resin [46]. Note, we also evaluated if the inactive Diaion-bound material might possibly have inhibitory activity by assessing its ability to inhibit LPS-induced cytokine production but found that this material was inactive, having no effect on LPS induced cytokine production (data not shown). Future chemical and physical analysis of these fractions will be necessary for characterization of Diaion-bound and unbound polysaccharide fractions and definition of the polysaccharide structures responsible for immunomodulatory activities.

Previously it was proposed that selective immunostimulatory and anti-inflammatory properties of citrus polysaccharides could be attributed to different degrees of methyl esterification [47-49]. In the present study, we found that the polysaccharide fraction with the most potent immunomodulatory activity (**CSP-AU1**) had a low degree of methyl esterification of uronic acid residues. Thus, free carboxylic groups and associated electrical charge may enhance the bioavailability and binding potential of **CSP-AU1** polysaccharides to different cell receptors.

*Clerodendrum* polysaccharides and recently characterized polysaccharides from *Alchornea cordofolia*[50] seem to be unique, as compared to LPS and many other plant polysaccharides, in their ability to induce GM-CSF production by MonoMac-6 cells and PBMCs. Indeed, previously characterized polysaccharide fractions **A-I** from *A. tripartite*[23], **T-I** from *T. vulgare*[24], **C-I** from *O. polyacanta*[25], and even high concentrations of LPS (up to 10 μg/ml) were unable to stimulate GM-CSF production by human PBMCs, whereas **CSP-AU, CSP-AU1, CSP-NU1**, and **CSP-NU2** all significantly induced production of this cytokine by MonoMac-6 cells and PBMCs.

Because Con A stimulated GM-CSF production by human PBMCs (Figure 5), we considered whether the GM-CSF-inducing activity of *Clerodendrum* polysaccharide fractions might correlate with hemagglutinating activity but found that the *Clerodendrum* polysaccharides, as well as polysaccharides isolated from *A. cordofolia*[50], had no hemagglutinating activity (up to 1 mg/ml). On the other hand, we found that polysaccharide **CP-AU** isolated from *Combretum racemosum* had relatively high hemagglutinating activity but minimally stimulated GM-CSF production by human PBMCs and only at the highest tested concentration (0.5 mg/ml) [51]. Thus, plant polysaccharide structures responsible for GM-CSF stimulation and hemagglutination appear to be distinct from each other.

Differences in intracellular signaling pathways were observed between *Clerodendrum* polysaccharides and previously reported immunomodulatory botanical polysaccharides. For example, polysaccharides from algae *Capsosiphon fulvescens* induced ERK1/2, but not p38 [52], polysaccharide **SP1** from *Caulerpa lentillifera* increased the phosphorylation of p38 [53], whereas polysaccharide **AP-1** from *Platycodon grandiflorum* activated phosphorylation of all three MAPKs [46]. Here, we demonstrated that **CSP-AU1** induces phosphorylation of Akt2, Akt3, GSK-3α/β, p38β/γ/δ, p70S6K1, RSK2, and mTOR, but not ERK1/2 in human peripheral blood mononuclear cells. Thus, it appears that various immunomodulatory polysaccharides exert their biological activities through different modes of action.

Plant polysaccharides can modulate leukocyte activity via different receptors, including complement receptor 3 (CR3), scavenger receptor, dectin-1, mannose receptor, galectin 3, and TLR4 (reviewed in [22]). For example, polysaccharides **ASP** and **G1-4A**, which were isolated from *Acanthopanax senticosus* and *Tinospora cordifolia*, respectively, activated macrophages and B cells by interacting with TLR4 [54,55]. TLR4 was also found to be one of the cellular receptors mediating TNF secretion induced by **ZPF1**, polysaccharide isolated from *Dioscorea batatas*[56]. Likewise, polysaccharide **CWSP** from *Chlorella pyrenoidosa* induced cytokine secretion in macrophages via TLR4-mediated signaling pathways [57]. In the present work, we found that the *Clerodendrum* polysaccharide sub-fraction **CSP-AU1** induced secretion of TNF in human PBMCs mainly via TLR4, but binding affinity for this TLR agonist was much lower than that of *E.coli*-derived LPS. Indeed, a 10-fold higher concentration of LPS-RS over LPS was necessary to completely inhibit LPS-induced TNF production in human PBMC. In comparison, a much lower relative concentration of LPS-RS was needed to inhibit **CSP-AU1** activity. Thus, the pharmacological properties of such low-affinity TLR4/MD-2 ligands may be different than those of bacterial LPS. Indeed, although the profiles of pro-inflammatory mediators (NO and most of the cytokines studied here) for *in vitro* stimulatory activity by bacterial LPS and **CSP-AU** were fairly similar (Figures 1, 2, 3, and 6), the profile was quite different for production of GM-CSF (Figure 4). Because several plant polysaccharides were recently found having therapeutic effects in different models of autoimmune diseases, such as arthritis [31,34], lupus erythematosus-like syndrome [30,33], autoallergic mouse model of Sjogren’s syndrome [58], asthma [59], and EAE [32], we suggest that such natural low-affinity TLR4 agonists may have clinically beneficial effects on autoimmune diseases by their induction of immunosuppression during chronic administration [29].

Although medical application of hot-water extracts from *C. splendens* for the treatment of autoimmune diseases has not been reported to date, in the present work we studied therapeutic effects of **CSP-AU1** in a mouse model of EAE. We found that chronic *i.p.* administration of **CSP-AU1** induced clinically beneficial effects in EAE, supporting the immunomodulatory properties of *Clerodendrum* polysaccharides *in vivo*. Our data showed that **CSP-AU1** treatment of mice with EAE delayed disease onset and reduced the clinical severity of EAE. Only one plant polysaccharide, ginsan from *Panax ginseng*, has been tested to date in this animal model of multiple sclerosis [32]. Similar to our results, these authors observed reduced EAE severity. The polysaccharide ginsan also has been reported to exhibit an anti-allergic reaction in an ovalbumin-induced murine asthma model [60]. Although various plant polysaccharides have been reported to have therapeutic activity in some other models of autoimmune diseases [30,31,33,34,58], mechanisms of immune suppression by these high-molecular weight molecules in autoimmune disease remain unresolved. Because different plant polysaccharides (e.g., [61]) and **CSP-AU1** activate cells, presumably via TLR4, it is possible that chronic stimulation of the innate immune system via TLR4 could lead to the induction of immunosuppression [29]. Indeed, Vaknin *et al.*[29] demonstrated that repetitive administration of LPS was sufficient to induce bystander T-cell immunosuppression. Moreover, LPS-stimulated dendritic cells induced tolerance against established collagen-induced arthritis [62].

In attempts to determine possible immunomodulatory mechanisms of EAE delay by **CSP-AU1**, we evaluated cytokine production by MOG-peptide reactivated cells in culture, after isolation from **CSP-AU1**- and PBS-treated EAE mice, and found that the beneficial effect of **CSP-AU1** administration was accompanied by reduced IFN-γ, IL-13, IL-17, TNF, GM-CSF and increased TGF-β production. Indeed, a review of the literature regarding immune mechanisms of multiple sclerosis and EAE showed that these cytokines play an important role in progression of EAE disease. In EAE, increased number of IL-17- and IFN-γ-producing cells in the spinal cord resulted from peripheral expansion of these cells after immunization with myelin-derived antigen (reviewed in [63]). During the EAE disease effector phase, GM-CSF was reported to sustain neuroinflammation via myeloid cells that infiltrated the central nervous system [64]. TNF produced by myeloid cells accelerated the onset of EAE disease by regulation of chemokine expression in the CNS, driving the recruitment of inflammatory cells into the target organ [65]. On the other hand, we recently found that regulatory T cells confer protection against EAE via TGF-β [19]. Thus, the higher level of TGF-β production by splenic and lymph node lymphocytes from **CSP-AU1**-treated EAE mice could be related to the beneficial therapeutic effects of this polysaccharide fraction.

## Conclusion

In this study, we elucidate the molecular mechanism of monocyte/macrophage activation by polysaccharides isolated from leaves of *C. splendens*. We found that Diaion HP-20-unbound polysaccharides from *C. splendens* were not cytotoxic and activated PBMCs and monocyte/macrophages, resulting in stimulation of NO and cytokine production. Our data demonstrated that *Clerodendrum* polysaccharide sub-fraction **CSP-AU1**, which was one of the most potent activators of cytokine production, activated PBMCs and monocyte/macrophages via TLR4 and signaling cascades involving Akt2/3, GSK-3α/β, p38β, p38δ, p38γ, p70S6K1, RSK2, mTOR, and AP-1/NK-κB. We also found that chronic *i.p.* administration of **CSP-AU1** induced clinically beneficial effects in EAE, an experimental model of multiple sclerosis, supporting the immunomodulatory properties of *Clerodendrum* polysaccharides *in vivo*. Thus, our data provide a molecular basis to explain at least part of the beneficial therapeutic effects for hot-water extracts of *C. splendens*, and suggest that the polysaccharides from *C. splendens* have potential for immune modulation.

## Competing interests

The authors declare that they have no competing interests concerning this article.

## Authors’ contributions

KK, IAS, SJ, DWP, MAJ, YSO, and MTQ designed experiments and evaluated results. KK, IAS, SJ, LNK, and DSK performed experiments. AY collected ethnopharmacological data. KK, IAS, and MTQ drafted the manuscript. KK, IAS, DWP, MAJ, YSO, and MTQ corrected the manuscript for publication. All authors read and approved the final manuscript.

## Pre-publication history

The pre-publication history for this paper can be accessed here:

http://www.biomedcentral.com/1472-6882/13/149/prepub
